# Acute Articular Rheumatism Secondary to Catarrhal Influenza

**Published:** 1882-01

**Authors:** H. A. Beres


### VIII.—ACUTE ARTICULAR RHEUMATISM SECONDARY TO CATARRHAL INFLUENZA.

REPORTED BY H. A. BERES, V. S.

November 7th, was called to attend a bay mare, aged six. She had been suffering from a mild attack of Catarrhal Influenza, from which she had been convalescing for several days.

Suddenly (a few hours previous to my first visit), she had been seized with stiffness; inability to move. I found all her joints swollen and inflamed, profuse diaphoresis, and symptoms of excruciating pain; pulse full and rapid. Diagnosticated Rheumatic Fever. Although in a comfortable stable and well cared for, she seemed the picture of agonized suffering. For forty-eight hours I followed the usual line of treatment with no abatement of symptoms.

While considering what next to do, I recalled a cure reported in the earlier number of the JOURNAL, and concluded I would try Salicin. Drachm doses were ordered every two hours, till the symptoms lessened. The result surpassed my expectations. In six hours the patient was free from pain; in ten hours she could lie down naturally and comfortably. The fever abated, the inflammation and swelling of the joints subsided, and in forty-eight hours she was apparently as well as ever. Salicin seemed to act so much like a specific in this case that I hope others may be induced to test its virtues as an anti-rheumatic remedy. It has been rather unusual in my experience for Acute Articular Rheumatism to follow Catarrhal Influenza, and I certainly have never had such magic results follow when I have prescribed other remedies in this disease.

BOSTON, Mass.

